# Enhancing Nurses’ Work Engagement Through Psychological Empowerment: Evidence from Saudi Arabia

**DOI:** 10.3390/healthcare14203406

**Published:** 2026-10-12

**Authors:** Wdad Alanazy, Waad Alenezi

**Affiliations:** 1 Department of Maternity and Child Health Nursing, College of Nursing, Majmaah University, Al-Majmaah 11952, Saudi Arabia; 2 Department of Nursing Administration, College of Nursing, Majmaah University, Al-Majmaah 11952, Saudi Arabia; 461204911@s.mu.edu.sa

**Keywords:** psychological empowerment, work engagement, nursing workforce, Saudi Arabia

## Abstract

**Background:** Nurses’ work engagement is an important determinant of nursing performance, staff well-being, and healthcare quality. Psychological empowerment may contribute to work engagement by strengthening nurses’ sense of meaning, competence, self-determination, and impact in their work. **Objective:** This study aimed to examine the relationship between psychological empowerment and work engagement among nurses working in the Second Health Cluster in Riyadh, Saudi Arabia. **Methods:** A cross-sectional study was conducted among nurses working in the Second Health Cluster. Data were collected using self-administered questionnaires measuring psychological empowerment and work engagement. Descriptive statistics, correlation analysis, and simple and multiple linear regression analyses were used to examine the associations between psychological empowerment, its dimensions, and work engagement. **Results:** The mean total psychological empowerment score was 41.48 ± 11.11 (possible range: 12–60), corresponding to a mean item score of 3.46 ± 0.93 on the 5-point response scale. Psychological empowerment was positively associated with work engagement in the simple regression model, which explained 57.0% of the variance in work engagement (R^2^ = 0.570, *p* < 0.001). In the multiple regression model, which included the four psychological empowerment dimensions, the explained variance was 57.5% (R^2^ = 0.575). Meaning (β = 0.293, *p* < 0.001), impact (β = 0.268, *p* < 0.001), and self-determination (β = 0.160, *p* = 0.016) were statistically significant independent correlates of work engagement, whereas competence was not statistically significant after adjustment for the other dimensions (β = 0.103, *p* = 0.117). **Conclusions:** Psychological empowerment was positively associated with nurses’ work engagement. Meaning, impact, and self-determination showed statistically significant associations when the four dimensions were examined simultaneously. These findings highlight the potential relevance of empowerment-related workplace practices while requiring cautious interpretation because of the cross-sectional design and sampling approach.

## 1. Introduction

Nurses constitute the largest group of healthcare professionals and are fundamental to delivering safe, effective, and patient-centered care. Their work engagement is closely associated with healthcare quality, patient safety, organizational performance, and workforce sustainability [1]. However, increasing healthcare demands, workforce shortages, heavy workloads, emotional stress, and limited professional autonomy place considerable psychological pressure on nurses, negatively affecting their motivation and engagement [2]. Reduced work engagement has been linked to burnout, turnover intention, and poorer patient outcomes, making the promotion of engagement a strategic priority for healthcare organizations [3]. Work engagement is defined as a positive, fulfilling work-related psychological state characterized by vigor, dedication, and absorption [4]. Engaged nurses demonstrate greater enthusiasm, resilience, and commitment to professional responsibilities, resulting in improved teamwork, enhanced quality of care, greater productivity, and higher patient satisfaction. Conversely, low engagement contributes to emotional exhaustion, reduced performance, and increased workforce instability [5,6].

A useful theoretical perspective for understanding factors that may contribute to nurses’ work engagement is Spreitzer’s Psychological Empowerment Theory [7]. Spreitzer conceptualized psychological empowerment as an intrinsic motivational state reflected in four cognitions: meaning, competence, self-determination, and impact [7,8]. Meaning refers to the perceived value and purpose of one’s work; competence reflects confidence in one’s ability to perform work activities effectively; self-determination represents a sense of autonomy and choice in initiating and regulating work-related actions; and impact refers to the perceived influence an individual has over organizational or work-related outcomes [9,10]. From this perspective, nurses who perceive their work as meaningful, feel competent in their professional roles, experience autonomy in decision-making, and believe that their actions have an impact may be more intrinsically motivated and more actively engaged in their work.

Creating supportive work environments has therefore become essential to sustaining nurses’ engagement. Organizational factors such as participative leadership, professional development opportunities, autonomy, and access to resources have been shown to strengthen nurses’ motivation and resilience [11]. Among these factors, empowerment has emerged as a key determinant of positive nursing outcomes. Empowerment encompasses both structural empowerment, which refers to organizational access to information, support, resources, and opportunities, and psychological empowerment, which reflects employees’ perceptions of meaning, competence, self-determination, and impact in their work [12,13].

Psychological empowerment is an intrinsic motivational construct that enhances nurses’ confidence, autonomy, and sense of purpose, thereby promoting greater professional commitment and engagement [14]. Previous studies have associated psychological empowerment with improved job satisfaction, creativity, work performance, and organizational commitment [15,16,17]. Despite growing international evidence, research examining the relationship between psychological empowerment and nurses’ work engagement in Saudi Arabia remains limited. Given ongoing healthcare transformation under Saudi Vision 2030, context-specific evidence is needed to better understand this relationship. Therefore, this study aimed to examine the association between psychological empowerment and work engagement among nurses working in Riyadh, Saudi Arabia.

## 2. Methods

**Study Design and Setting:** A descriptive cross-sectional correlational study was conducted among registered nurses working in the Second Health Cluster, Riyadh, Saudi Arabia. The Second Health Cluster comprises multiple hospitals and specialized healthcare facilities providing primary, secondary, and tertiary healthcare services.

**Participants and Sampling:** Registered nurses who had at least six months of clinical experience in the Second Health Cluster and provided informed consent were eligible to participate. Nurse managers and administrative nurses were excluded because their primary roles involve managerial, supervisory, administrative, or organizational responsibilities rather than direct clinical nursing practice. These role differences may influence perceptions of empowerment and work engagement through differences in job responsibilities, decision-making authority, organizational influence, and exposure to clinical practice. Including managerial and administrative nursing personnel could therefore introduce substantial heterogeneity into the study population and make it more difficult to interpret empowerment and engagement specifically among nurses engaged in direct patient care. Nurses who were on leave during the data collection period and those with less than six months of employment were also excluded. Nurses with less than six months of employment were excluded to ensure that participants had sufficient experience within their current clinical environment to provide informed responses regarding workplace empowerment and engagement.

A non-probability convenience sampling method was used to recruit eligible nurses from participating hospitals and clinical units within the Second Health Cluster. Eligible nurses who were accessible during the data collection period were invited to participate voluntarily. No random sampling or proportional allocation across hospitals, clinical units, or nursing subgroups was performed. Based on a 95% confidence level and a 5% margin of error, the minimum required sample size was calculated to be 384 nurses. To account for potential non-response and incomplete questionnaires, a target sample of 400 nurses was recruited.

**Measures:** Data were collected using a structured self-administered questionnaire consisting of three sections: demographic and professional characteristics, including age, gender, nationality, educational level, years of experience, work unit, employment status, shift pattern, and length of service; the Utrecht Work Engagement Scale (UWES-9) [18], a validated nine-item instrument measuring three dimensions of work engagement, vigor, dedication, and absorption, in which items are rated on a seven-point Likert scale, with higher scores indicating greater work engagement; and the Psychological Empowerment Scale (PES) [7], a validated 12-item instrument assessing four dimensions, meaning, competence, self-determination, and impact, in which items are rated on a five-point Likert scale, with higher scores indicating greater psychological empowerment.

Before conducting the main study, a pilot study was conducted among a group of randomly selected nurses to assess the clarity, content, flow, and linguistic appropriateness of the questionnaire. The reliability of the study instruments was assessed using Cronbach’s alpha. The Cronbach’s alpha coefficient was 0.916 for the UWES-9 and 0.942 for the PES, indicating excellent internal consistency and supporting the reliability of the instruments for use in the main study. Nurses who participated in the pilot study were excluded from the main study sample.

**Data Collection:** Data were collected over four months from May to August 2026, using an anonymous online questionnaire developed in Google Forms. The survey link was distributed through official hospital communication channels. Participation was voluntary, and electronic informed consent was obtained before questionnaire completion.

**Data Analysis:** Data were analyzed using IBM SPSS Statistics version 26. Descriptive statistics (frequencies, percentages, means, and standard deviations) summarized participant characteristics and study variables. Pearson’s correlation coefficient examined the association between psychological empowerment and work engagement. Independent-sample *t*-tests and one-way analysis of variance (ANOVA) compared mean scores across demographic groups. Multiple linear regression analysis was performed to determine whether psychological empowerment and its dimensions predicted nurses’ work engagement. Statistical significance was set at *p* < 0.05.

**Ethical Considerations:** Ethical approval for this study was obtained from the Institutional Review Board (IRB) of Majmaah University and the Riyadh Second Health Cluster prior to the commencement of data collection (IRB No. 26-288C). Participation was voluntary, and all participants provided electronic informed consent before accessing and completing the questionnaire. Participants were informed about the purpose of this study, the voluntary nature of their participation, and their right to withdraw from this study at any time without penalty. Confidentiality and anonymity were maintained throughout this study, and no personally identifying information was collected or disclosed. All collected data were securely handled and used solely for research purposes.

## 3. Result

This study included 400 registered nurses working in the Second Health Cluster, Riyadh. The results describe participants’ characteristics, levels of psychological empowerment and work engagement, associations with demographic and work-related characteristics, and the relationship between psychological empowerment and work engagement.

### 3.1. Participant Characteristics

Table 1 presents the demographic and work-related characteristics of the study sample (n = 400). Regarding age, 182 participants (45.5%) were aged 31–40 years, followed by 111 (27.8%) aged 25–30 years. The sample was predominantly female, with 363 participants (90.8%) being female and 37 (9.2%) being male. Most participants were Saudi nationals (n = 390, 97.5%). Regarding educational level, 137 participants (34.3%) held a Bachelor of Science in Nursing (BSN), followed by 82 (20.5%) with a master’s degree. Regarding nursing experience, 153 participants (38.3%) had 6–10 years of experience, followed by 121 (30.3%) with 2–5 years of experience. The Medical/Surgical Ward was the most represented clinical setting (n = 127, 31.8%), followed by the Intensive Care Unit (ICU) (n = 111, 27.8%). Most participants were employed full-time (n = 251, 62.8%), and the most common shift pattern was a fixed night shift (n = 131, 32.8%).

### 3.2. Perceived Level of Psychological Empowerment

Table 2 presents the descriptive statistics for psychological empowerment and its four dimensions. The overall mean psychological empowerment score was 3.46 (SD = 0.93), corresponding to 61.4% of the maximum possible score. Among the four dimensions, meaning had the highest mean score (M = 3.52, SD = 1.01), whereas impact had the lowest (M = 3.41, SD = 0.98).

### 3.3. Perceived Level of Work Engagement

Table 3 presents the descriptive statistics for work engagement and its three dimensions. The overall mean work engagement score was 3.34 (SD = 1.29), corresponding to 55.6% of the maximum possible score. Among the three dimensions, absorption had the highest mean score (M = 3.44, SD = 1.39), followed by dedication (M = 3.30, SD = 1.45) and vigor (M = 3.26, SD = 1.41).

### 3.4. Association Between Study Variables

Table 4 presents the associations between participants’ demographic and work-related characteristics and total psychological empowerment and total work engagement. Independent-sample *t*-tests were used for variables with two categories, whereas one-way analysis of variance (ANOVA) was used for variables with more than two categories. Statistically significant differences in total work engagement were observed according to gender, age group, educational level, current clinical unit, shift rotation, and years of nursing experience (all *p* < 0.05). For psychological empowerment, statistically significant differences were observed according to gender, age group, educational level, current clinical unit, shift rotation, years of nursing experience, and nationality (*p* = 0.049). Employment status was not significantly associated with either work engagement or psychological empowerment.

For the two-group comparisons, gender was significantly associated with work engagement, t(398) = 6.553, *p* < 0.001, and psychological empowerment, t(398) = 4.223, *p* < 0.001. Employment status was not significantly associated with work engagement, t(398) = −0.336, *p* = 0.740, or psychological empowerment, t(398) = 1.827, *p* = 0.070. Nationality was not significantly associated with work engagement, t(398) = −1.402, *p* = 0.162, whereas the difference in psychological empowerment was statistically significant, t(398) = −1.974, *p* = 0.049. For variables with more than two categories, statistically significant differences were observed across age groups, educational levels, current clinical units, shift rotations, and years of nursing experience for both work engagement and psychological empowerment (Table 4).

Table 5 demonstrates that all four dimensions of psychological empowerment were positively and significantly correlated with all dimensions of work engagement (all *p* < 0.001). Correlation coefficients ranged from 0.616 to 0.705 for the individual psychological empowerment dimensions and from 0.684 to 0.755 for total psychological empowerment. The strongest association was observed between total psychological empowerment and total work engagement (r = 0.755, *p* < 0.001), indicating a strong positive correlation between the two constructs.

Table 6 presents the simple linear regression analysis examining the association between total psychological empowerment and work engagement. Total psychological empowerment was positively and significantly associated with work engagement (β = 0.755, t = 22.970, *p* < 0.001). The model explained 57.0% of the variance in work engagement (R^2^ = 0.570; adjusted R^2^ = 0.569) and was statistically significant, F(1398) = 527.500, *p* < 0.001.

#### Multiple Linear Regression Analysis

A multiple linear regression analysis was conducted to examine the independent associations of the four psychological empowerment dimensions—meaning, competence, self-determination, and impact—with total work engagement (Table 7). The overall regression model was statistically significant, F(4395) = 133.480, *p* < 0.001, and explained 57.5% of the variance in total work engagement (R^2^ = 0.575; adjusted R^2^ = 0.570). After adjustment for the other psychological empowerment dimensions, meaning was positively and significantly associated with work engagement (β = 0.293, *p* < 0.001; 95% CI for B: 0.221–0.528). Impact was also positively and significantly associated with work engagement (β = 0.268, *p* < 0.001; 95% CI for B: 0.184–0.517). Self-determination showed a statistically significant positive association with work engagement (β = 0.160, *p* = 0.016; 95% CI for B: 0.038–0.366). In contrast, competence was not statistically significant after adjustment for the other three dimensions (β = 0.103, *p* = 0.117; 95% CI for B: −0.033–0.296).

Tolerance values ranged from 0.247 to 0.289, while variance inflation factor (VIF) values ranged from 3.462 to 4.042. These results indicate overlap among the psychological empowerment dimensions, which is consistent with their conceptual and statistical relationships. Although the VIF values were below commonly used thresholds for severe multicollinearity, the observed overlap warrants caution when interpreting the individual regression coefficients. Accordingly, the coefficients should be understood as the unique associations of each dimension with work engagement after accounting for the other dimensions.

## 4. Discussion

This study examined psychological empowerment (PE), work engagement (WE), their relationship, and the association between PE and WE among nurses working in the Second Health Cluster in Riyadh. Overall, nurses reported relatively higher levels of psychological empowerment and work engagement, with variation across the four empowerment dimensions and three engagement dimensions. A strong positive relationship was identified between psychological empowerment and work engagement. These findings are consistent with Social Cognitive Theory and Structural Empowerment Theory, suggesting that nurses who perceive their work as meaningful, feel competent in their roles, experience greater autonomy, and perceive greater influence within their work environment may report higher levels of work engagement.

Psychological empowerment was relatively high, indicating that nurses generally perceived their roles as meaningful and valued within the healthcare system. This finding is consistent with previous Saudi studies that reported moderate-to-high empowerment among nurses and linked these outcomes to ongoing healthcare reforms and professional development initiatives under Saudi Vision 2030 [19,20]. Such organizational and professional developments may provide nurses with greater opportunities to participate in patient care, professional development, and organizational improvement. However, because the present study used a cross-sectional design, these contextual factors cannot be established as direct explanations for the observed empowerment levels.

Among the four dimensions of psychological empowerment, meaning received the highest score, suggesting that nurses generally viewed their work as personally important and aligned with their professional values. This finding agrees with previous research indicating that meaning is an important component of psychological empowerment because it connects individual values with professional and organizational goals [8]. Competence also received a relatively high score, indicating that participants generally perceived themselves as capable of performing their clinical responsibilities. This finding may be consistent with the professional experience and educational background represented in the study sample; however, the cross-sectional design does not allow a causal relationship between these characteristics and perceived competence to be established.

In contrast, self-determination and impact received comparatively lower scores. These findings may indicate that, although nurses viewed their work as meaningful and felt capable of performing their clinical roles, they perceived comparatively less autonomy in determining how work was performed and less influence over organizational decisions. The hierarchical structure of healthcare organizations and the presence of institutional policies and established clinical decision-making processes may contribute to such perceptions, although these factors were not directly measured in the present study. Previous Saudi studies have similarly reported concerns regarding professional autonomy among nurses [21]. The findings therefore highlight the importance of creating organizational mechanisms through which nurses can participate more directly in decisions affecting clinical practice, quality improvement, and their work environment.

Work engagement was also relatively high, although differences were observed across its dimensions. The findings should be interpreted within the organizational context of the Second Health Cluster, which encompasses multiple healthcare facilities and clinical environments with differing workloads, staffing requirements, leadership structures, patient-care demands, and opportunities for professional participation. Such structural differences may shape nurses’ daily work experiences and opportunities to exercise autonomy, access resources, and remain engaged in their roles. Rather than attributing engagement to a single organizational factor, the findings suggest that work engagement may reflect the interaction of individual resources with characteristics of the work environment. Previous studies conducted in Saudi Arabia have similarly reported varying levels of work engagement among nurses [8,22,23].

Among the three engagement dimensions, absorption received the highest score, indicating that participants generally reported substantial concentration and psychological involvement in their work. This finding suggests that nurses were able to maintain attention and become deeply involved in their professional activities. The relatively higher absorption score is consistent with the overall pattern of work engagement observed in the present study and highlights the importance of cognitive involvement in nurses’ professional roles.

This study demonstrated a strong positive relationship between psychological empowerment and work engagement, with higher empowerment consistently associated with greater engagement. This finding supports the Job Demands–Resources (JD-R) model, which proposes that personal and organizational resources can facilitate motivation and work engagement. Similar positive associations have been reported among Saudi nurses by Al Otaibi et al. (2023), Abualruz et al. (2024), and Alotaibi et al. (2022) [8,23,24]. The correlation observed in the present study (r = 0.755) indicates a strong association between the two constructs. However, the magnitude of this correlation should not be interpreted as evidence that psychological empowerment causes higher work engagement, particularly because this study was cross-sectional and both constructs were measured at the same time.

The regression analysis demonstrated a positive association between psychological empowerment and work engagement. In the simple regression model, psychological empowerment explained 57.0% of the variance in work engagement (R^2^ = 0.570, *p* < 0.001). When the four dimensions of psychological empowerment were entered simultaneously into the multiple regression model, the explained variance was 57.5% (R^2^ = 0.575). Meaning, impact, and self-determination were statistically significant independent correlates of work engagement, whereas competence did not retain statistical significance after adjustment for the other dimensions. Although competence was positively associated with work engagement in the bivariate analysis (r = 0.679, *p* < 0.001), its adjusted coefficient was not statistically significant (β = 0.103, *p* = 0.117). This difference may reflect shared variance among the psychological empowerment dimensions, particularly because the dimensions were moderately to strongly correlated. The tolerance and VIF results also indicate some overlap among the predictors. However, these findings do not establish that multicollinearity alone explains the nonsignificant adjusted coefficient. Rather, the result suggests that competence did not demonstrate a statistically significant unique association with work engagement when the other empowerment dimensions were considered simultaneously. These findings support interpreting psychological empowerment as a multidimensional construct and distinguishing its overall association with work engagement from the independent contribution of each dimension.

Significant differences were observed across several demographic and work-related characteristics. Older and more experienced nurses reported higher levels of psychological empowerment and work engagement. These findings are consistent with the possibility that professional experience may be associated with greater confidence, familiarity with organizational processes, and opportunities to exercise professional influence. However, because the present study was cross-sectional, these differences cannot establish that experience itself produces greater empowerment or engagement. Similarly, differences across educational groups may reflect differences in professional roles, responsibilities, opportunities, or other characteristics that were not independently examined in this study.

Nationality also warrants consideration when interpreting the findings. In the present study, psychological empowerment differed significantly by nationality, whereas the difference in work engagement was not statistically significant. Because the nursing workforce in Saudi healthcare settings includes Saudi and expatriate nurses, nationality may be associated with differences in cultural background, previous professional experiences, familiarity with the local healthcare system, communication patterns, organizational integration, and perceptions of autonomy and influence. However, nationality should not be interpreted as a direct cause of differences in empowerment. Rather, it may serve as a marker for broader social, cultural, professional, and organizational experiences that were not directly measured in this study. These findings highlight the importance of considering workforce diversity when developing organizational approaches to psychological empowerment.

Differences were also observed across clinical units, which may reflect variation in workload, staffing, leadership, teamwork, professional autonomy, and resource availability across clinical environments. These findings are consistent with the JD-R framework, which emphasizes the importance of organizational resources in supporting employee motivation and engagement. Day-shift nurses also reported significantly higher empowerment and engagement than night-shift nurses. Differences in access to supervision, professional development opportunities, staffing patterns, fatigue, and work schedules may contribute to this pattern, although these factors were not directly measured in the present study [25]. Future research should examine these potential mechanisms more directly.

### 4.1. Implications for Nursing Practice

The findings have several practical implications for nursing leadership and workforce management. Rather than relying on broad empowerment initiatives, healthcare organizations could implement specific structures that provide nurses with opportunities to exercise autonomy, participate in decision-making, and influence clinical practice. For example, nursing leadership could establish or strengthen unit-based shared governance councils in which staff nurses have defined representation and participate in decisions related to staffing processes, clinical policies, patient-safety initiatives, and quality-improvement priorities. Each council could use a formal agenda, document recommendations, and provide feedback to nursing management regarding which recommendations were adopted and why. This approach would provide nurses with a structured mechanism through which the dimensions of autonomy and impact can be translated into routine organizational practice.

Clinical ladder programs could also be implemented or strengthened to provide nurses with clearly defined opportunities for professional advancement. Such programs could include transparent competency levels, defined clinical and leadership competencies, participation in evidence-based practice or quality-improvement projects, and recognition of progression through the clinical ladder. Linking advancement to demonstrable professional competencies and contributions to clinical improvement may help strengthen nurses’ perceptions of competence and professional influence.

For less experienced nurses, structured mentorship programs could provide another practical empowerment-focused strategy. A formal program could pair newly appointed or less experienced nurses with experienced nurse mentors for a defined period, such as the first 6–12 months of employment. Mentorship could include scheduled meetings, clinical goal setting, case discussions, feedback on professional development, and guidance regarding organizational processes and available professional resources. Such programs could be particularly relevant for nurses who report lower confidence or limited familiarity with organizational decision-making processes.

Nursing leaders could also provide nurses with formal opportunities to participate in quality-improvement and evidence-based practice projects. For example, each clinical unit could identify specific patient-care or workflow problems and invite staff nurses to participate in project teams responsible for assessing the problem, developing interventions, monitoring outcomes, and presenting results to nursing leadership. Providing nurses with responsibility for defined components of these projects may strengthen their sense of competence and influence over clinical practice.

Given the lower levels of empowerment and engagement observed among some work-related groups, particularly nurses with less experience and those working night shifts, interventions could also be tailored to these groups. Night-shift nurses could be provided with equivalent access to professional development, leadership communication, mentorship, and quality-improvement opportunities rather than limiting such activities to daytime staff. Similarly, managers could conduct periodic unit-level empowerment assessments and use the findings to identify areas requiring improvement in autonomy, participation, recognition, and access to resources.

Importantly, these strategies should be accompanied by measurable implementation outcomes. Nursing leadership could monitor indicators such as participation in shared governance activities, completion of mentorship programs, clinical ladder progression, nurse participation in quality-improvement projects, and changes in empowerment and work engagement scores over time. Such monitoring would allow organizations to determine whether empowerment-focused initiatives are being implemented as intended and whether changes in nurses’ perceptions and work experiences occur following implementation.

Overall, the findings suggest that psychological empowerment is an important correlate of nurses’ work engagement. Organizational efforts should therefore move beyond general recommendations to promote empowerment and instead create specific mechanisms through which nurses can exercise autonomy, develop professional competence, participate in decision-making, and influence clinical practice. Shared governance councils, clinical ladder programs, structured mentorship, and nurse-led quality-improvement initiatives represent practical approaches that could be considered within the Second Health Cluster and evaluated through future longitudinal research.

### 4.2. Limitations

This study has several limitations that should be considered when interpreting the findings. First, the cross-sectional design precludes causal inferences regarding the relationship between psychological empowerment and work engagement. Although psychological empowerment was significantly associated with work engagement in the regression analysis, the cross-sectional design does not establish temporal or causal relationships between the variables. Second, both psychological empowerment and work engagement were assessed using self-reported measures collected at a single time point. This may introduce common method variance, whereby correlations between variables may be influenced partly by the use of the same measurement method. In addition, responses may be affected by social desirability bias, particularly when participants are reporting perceptions of their workplace, autonomy, and engagement. Future studies could incorporate longitudinal designs and, where feasible, multiple data sources or objective indicators to reduce these potential sources of bias.

Third, convenience sampling was used to recruit participants from a single hospital. Although this approach facilitated recruitment within the study setting, participants were not randomly selected from a defined sampling frame. Consequently, selection bias cannot be excluded, and the findings should be interpreted with caution when considering their generalizability to nurses working in other hospitals, regions, or healthcare settings in Saudi Arabia. Another limitation is that nurse managers and administrative nurses were excluded because this study focused on nurses primarily engaged in direct clinical practice. Consequently, the findings may not reflect the experiences of nurses working in managerial, supervisory, or administrative roles, whose levels and sources of empowerment and work engagement may differ because of their distinct responsibilities and decision-making authority. Future studies could include both clinical and managerial nursing personnel to provide a more comprehensive assessment of empowerment and engagement across different nursing roles within the health cluster. In addition, although nationality was examined as a demographic characteristic, this study did not comprehensively assess cultural background, migration status, length of employment in Saudi Arabia, country of professional training, language-related factors, or experiences of organizational and cultural integration. These factors may influence perceptions of autonomy, self-determination, organizational influence, and work engagement among a culturally diverse nursing workforce. Therefore, the observed difference in psychological empowerment by nationality should be interpreted cautiously and should not be assumed to represent a direct cultural effect. Future studies should examine these factors more comprehensively and consider multivariable analyses that account for relevant demographic, professional, and organizational characteristics.

Finally, other factors that may be associated with work engagement, such as leadership, organizational support, burnout, job satisfaction, emotional intelligence, and professional identity, were not examined. Their omission may limit the comprehensiveness of the explanatory model. Future research should employ longitudinal or mixed-method designs and incorporate these variables to provide a more comprehensive understanding of the factors associated with nurses’ work engagement.

## 5. Conclusions

The findings indicate that psychological empowerment was positively associated with work engagement among nurses working in the Second Health Cluster in Riyadh, Saudi Arabia. When the four dimensions of psychological empowerment were examined simultaneously, meaning, impact, and self-determination were statistically significant independent correlates of work engagement, whereas competence was not statistically significant after adjustment for the other dimensions. These findings suggest that nurses’ perceptions of the meaning and impact of their work, together with their sense of self-determination, may be relevant to work engagement. Healthcare organizations within the studied cluster, and potentially those operating within similar regional healthcare frameworks, may consider workplace practices that support nurses’ autonomy, meaningful participation, and perceived contribution to organizational and patient-care outcomes. However, the cross-sectional design precludes causal conclusions, and the use of non-probability convenience sampling within a single healthcare cluster limits the generalizability of the findings to the wider nursing population of Saudi Arabia. Therefore, these findings and organizational implications should be interpreted primarily in the context of the studied cluster and similar settings. Further research involving nurses from diverse healthcare clusters and regions, using more representative sampling approaches, is warranted to examine these relationships and assess the applicability of potential organizational strategies across different healthcare settings.

## Figures and Tables

**Table 1:** Demographic and Work-Related Characteristics of the Participants (n = 400).

Socio-Demographic and Work-Related Characteristics (n = 400)		
Category	n	%
Age Group		
31–40 years	182	45.5
25–30 years	111	27.8
Less than 25 years	55	13.8
More than 40 years	52	13.0
Gender		
Female	363	90.8
Male	37	9.2
Nationality		
Saudi	390	97.5
Non Saudi	10	2.5
Educational Level		
Bachelor of Science in Nursing (BSN)	137	34.3
Master’s Degree (MSc Nursing)	82	20.5
Postgraduate Diploma	80	20.0
Diploma in Nursing	59	14.8
Doctorate (PhD/DNP)	42	10.5
Years of Experience		
6–10 years	153	38.3
2–5 years	121	30.3
More than 10 years	88	22.0
Less than 2 years	38	9.5
Current Unit		
Medical/Surgical Ward	127	31.8
Intensive Care Unit (ICU)	111	27.8
Emergency Department	82	20.5
Other	80	20.0
Employment Status		
Full time	251	62.8
Part-time	149	37.3
Shift Rotation		
Fixed Night shift	131	32.8
Fixed day shift	126	31.5
Rotating shifts (day & night)	116	29.0
Flexible/on-call shifts	27	6.8

**Table 2:** Descriptive Statistics for Psychological Empowerment and Its Dimensions (N = 400).

Scale	Mean	SD	Min	Max	Total Possible Score	Mean %
Psychological Empowerment (Overall)	3.46	0.93	1	5	1–5	61.4%
Meaning	3.52	1.01	1	5	1–5	62.9%
Competence	3.49	1.01	1	5	1–5	62.1%
Self-determination	3.42	1.02	1	5	1–5	60.5%
Impact	3.41	0.98	1	5	1–5	60.1%
Total scale score	41.48	11.11	12	60	12–60	61.4%

**Table 3:** Descriptive Statistics for Work Engagement and Its Dimensions (N = 400).

Scale	Mean	SD	Min	Max	Total Possible Score	Mean %
Work Engagement (Overall)	3.34	1.29	0	6	0–6	55.6%
Vigor	3.26	1.41	0	6	0–6	54.3%
Dedication	3.30	1.45	0	6	0–6	55.0%
Absorption	3.44	1.39	0	6	0–6	57.4%
Total scale score	30.02	11.63	0	54	0–54	55.6%

**Table 4:** Differences in total psychological empowerment and total work engagement according to demographic and work-related characteristics (N = 400).

Characteristic	Total WE, Mean ± SD	Test Statistic (*p*)	Total PE, Mean ± SD	Test Statistic (*p*)
Gender				
Male	4.24 ± 0.82	t(398) = 6.553, *p* < 0.001	4.06 ± 0.68	t(398) = 4.223, *p* < 0.001
Female	3.24 ± 1.30		3.40 ± 0.93	
Age group				
<25 years	2.74 ± 1.56	F(3396) = 6.718, *p* < 0.001	2.88 ± 1.21	F(3396) = 13.664, *p* < 0.001
25–30 years	3.21 ± 1.49		3.30 ± 0.98	
31–40 years	3.49 ± 1.05		3.65 ± 0.71	
>40 years	3.68 ± 1.00		3.73 ± 0.78	
Educational level				
Diploma in Nursing	3.03 ± 1.48	F(4395) = 3.267, *p* = 0.010	3.13 ± 1.13	F(4395) = 3.885, *p* < 0.001
Postgraduate Diploma	3.06 ± 1.34		3.33 ± 0.96	
BSN	3.39 ± 1.36		3.51 ± 0.92	
Master’s (MSc)	3.57 ± 0.82		3.57 ± 0.65	
Doctorate (PhD/DNP)	3.67 ± 1.25		3.76 ± 0.82	
Employment status				
Full-time	3.32 ± 1.15	t(398) = −0.336, *p* = 0.740	3.53 ± 0.83	t(398) = 1.827, *p* = 0.070
Part-time	3.37 ± 1.50		3.34 ± 1.07	
Nationality				
Saudi	3.32 ± 1.30	t(398) = −1.402, *p* = 0.162	3.44 ± 0.93	t(398) = −1.974, *p* = 0.049
Non-Saudi	3.90 ± 1.08		4.03 ± 0.78	
Current clinical unit				
Emergency Department	3.13 ± 1.62	F(3396) = 5.606, *p* < 0.001	3.18 ± 1.15	F(3396) = 6.057, *p* < 0.001
ICU	3.15 ± 1.25		3.42 ± 0.80	
Medical/Surgical Ward	3.31 ± 1.03		3.46 ± 0.76	
Other	3.84 ± 1.20		3.79 ± 0.97	
Shift rotation				
Fixed day shift	3.56 ± 1.39	F(3396) = 11.634, *p* < 0.001	3.59 ± 1.00	F(3396) = 11.521, *p* < 0.001
Fixed night shift	2.82 ± 1.21		3.09 ± 0.91	
Flexible/on-call	3.39 ± 1.54		3.56 ± 1.10	
Rotating shifts	3.67 ± 0.99		3.71 ± 0.64	
Years of nursing experience				
<2 years	2.99 ± 1.52	F(3396) = 8.946, *p* < 0.001	3.26 ± 1.06	F(3396) = 8.993, *p* < 0.001
2–5 years	3.01 ± 1.44		3.17 ± 1.00	
6–10 years	3.38 ± 1.05		3.55 ± 0.76	
>10 years	3.86 ± 1.15		3.78 ± 0.89	

**Note.** WE = work engagement; PE = psychological empowerment; BSN = Bachelor of Science in Nursing; ICU = intensive care unit. Independent-sample *t*-tests were used for variables with two categories, and one-way ANOVA was used for variables with more than two categories. Statistical significance was set at *p* < 0.05.

**Table 5:** Correlation between staff nurses’ perception of psychological empowerment dimensions and work engagement dimensions (n = 400).

Psychological Empowerment		Work Engagement (UWES-9)			
Dimension		Vigor	Dedication	Absorption	Total WE
Meaning	***r***	0.616	0.639	0.670	0.703
	***p***	<0.001	<0.001	<0.001	<0.001
Competence	***r***	0.621	0.617	0.618	0.679
	***p***	<0.001	<0.001	<0.001	<0.001
Self-Determination	***r***	0.616	0.620	0.643	0.687
	***p***	<0.001	<0.001	<0.001	<0.001
Impact	***r***	0.661	0.624	0.644	0.705
	***p***	<0.001	<0.001	<0.001	<0.001
Total PE	***r***	0.684	0.681	0.701	**0.755**
	***p***	<0.001	<0.001	<0.001	**<0.001**

Note. WE = Work Engagement; PE = Psychological Empowerment. All correlations are statistically significant at *p* < 0.001.

**Table 6:** Simple linear regression analysis predicting work engagement from psychological empowerment (n = 400).

Predictor	B	SE	β	t	*p*
Constant	−0.307	0.164	—	−1.87	0.062
Total Psychological Empowerment	1.054	0.046	0.755	22.97	<0.001

**Model summary:** R = 0.755; R^2^ = 0.570; adjusted R^2^ = 0.569; F(1398) = 527.50; *p* < 0.001. **Note**. B = unstandardized regression coefficient; SE = standard error; β = standardized regression coefficient; R^2^ = coefficient of determination.

**Table 7:** Multiple linear regression analysis of psychological empowerment dimensions predicting total work engagement (n = 400).

Predictor	B	SE	β	t	*p*	95% CI for B	Tolerance	VIF
Constant	−0.327	0.164	—	−1.987	0.048	−0.650 to −0.003	—	—
Meaning	0.375	0.078	0.293	4.799	<0.001	0.221 to 0.528	0.289	3.462
Competence	0.132	0.084	0.103	1.571	0.117	−0.033 to 0.296	0.250	4.007
Self-determination	0.202	0.083	0.160	2.424	0.016	0.038 to 0.366	0.247	4.042
Impact	0.351	0.085	0.268	4.144	<0.001	0.184 to 0.517	0.258	3.877

**Model summary:** R = 0.758; R^2^ = 0.575; adjusted R^2^ = 0.570; F(4395) = 133.480, *p* < 0.001. **Note.** B = unstandardized regression coefficient; SE = standard error; β = standardized regression coefficient; CI = confidence interval; VIF = variance inflation factor.

## Data Availability

The datasets used or analyzed during this study are available from the corresponding author upon acceptable request due to privacy concern.
